# Improving Self-Healing and Shrinkage Reduction of Cementitious Materials Using Water-Absorbing Polymer Microcapsules

**DOI:** 10.3390/ma15030847

**Published:** 2022-01-23

**Authors:** Qianjin Mao, Jiayi Chen, Wenjing Qi, Hui Liu, Ziming Wang, Suping Cui

**Affiliations:** 1 Faculty of Materials and Manufacturing, Beijing University of Technology, Beijing 100124, China; chenjiayi@emails.bjut.edu.cn (J.C.); zenggd@emails.bjut.edu.cn (W.Q.); wangziming@bjut.edu.cn (Z.W.); cuisuping@bjut.edu.cn (S.C.); 2 Key Laboratory of Advanced Functional Materials, Ministry of Education of China, Beijing University of Technology, Beijing 100124, China; 3 Beijing Institute of Housing and Urban Rural Construction Science and Technology, Beijing 100021, China; liuhui9516@bjut.edu.cn

**Keywords:** autogenous shrinkage, self-healing, concrete, super absorb polymer, microcapsules

## Abstract

Self-healing cementitious materials are a promising means for ensuring sustainable concrete infrastructure and promoting long-term service lives. To obtain microcapsules that are versatile in varying environments, in this study, absorbing microcapsules with calcium alginate as the shell and epoxy resin as the core were prepared. The absorbing microcapsules exhibit self-healing and can reduce the shrinkage of cementitious materials. Volume changes of the microcapsules in the hardened paste with increasing hydration age were observed using three-dimensional X-ray computed tomography. In the hardened cement paste with a water-cement ratio of 0.29, the absorption of the microcapsules lasted for several days, and the release of water lasted for 28 days. The absorption of microcapsules affected the fluidity of cement paste, and it was significantly weakened and delayed due to the lower absorption rate. The addition of absorbing microcapsules significantly reduced the autogenous and drying shrinkage of mortars. For microcapsules with a core content of 55% added at 3.5% of cement weight, autogenous shrinkage was almost eliminated. Most importantly, the addition of absorbing microcapsules could achieve a certain degree of recovery of compressive strength as well as satisfactory recovery of impermeability in dry and wet environments.

## 1. Introduction

Concrete is prone to cracks in the process of construction and service, which has a great impact on the durability of concrete, considerably shortening its service life [1] and leading to high costs for the repair of structures [2]. Therefore, different methods are used to try to solve or inhibit the cracking of concrete, such as increasing humidity during curing to reduce cracking, using shrinkage reducing agents, and reducing the temperature difference between the inside and outside of the concrete. Furthermore, the concept of self-healing concrete is proposed to reduce the maintenance and repair of concrete structures in the use phase [3].

Self-healing concrete, which was inspired by the self-repairing phenomenon in biology, can extend the service lifetime of structures. While the idea remains a novelty in practice, it has attracted widespread attention in the research community. Many innovative approaches and strategies have been investigated to impart self-healing abilities to concrete [4,5]. These approaches aim toward improve autogenous cracks healing or modifying concrete by embedding capsules with healing agents to autonomously heal cracks. Polymeric capsules, as proposed by White et al. [6], are efficient in crack healing, and many studies have exhibited full or partial recovery of mechanical strength [7]. 

Snoeck et al. reported that superabsorbent polymers (SAPs) promoted the autogenous healing of cracks in concrete [8]. Superabsorbent polymers (SAPs) as internal curing agents for concrete can be used to inhibit the early cracking of concrete caused by shrinkage [9]. SAPs are a type of hydrogel formed by cross-linking of water-soluble polymers, and they can absorb water equivalent to hundreds of times of their own weight from the surrounding environment [10]. Li et al. [11] added SAP in cement; the autogenous shrinkage of cement decreased with increasing SAP content. Wang et al. [12] found that adding 0.05–0.1% SAPs to alkalized active slag mortar reduced autogenous shrinkage by 4–6 times. Asmann et al. [13] found that the shrinkage strain of mortar with a water-to-cement ratio of 0.42 decreased from 85 to 35 μstrain on addition of SAPs. Kong et al. [14] found that the addition of presoaked SAPs considerably decreased autogenous shrinkage as well as the early-age shrinkage of high-strength concrete under dry conditions. SAPs also decreased the total shrinkage and drying shrinkage of ultrahigh-strength concrete [15]. SAPs have some other advantages, such as regulating the pore structure [16], increasing impermeability [17], and freeze-thaw [18]. Moreover, SAPs have been found to promote the autogenous healing of microcracks [19]. In concrete, first, SAPs absorb water and expand when water enters a crack, and then, the expanded SAPs seal the crack, thus preventing harmful ions from entering the matrix [20]. Second, under dry conditions, the water absorbed by the SAPs is released to the surfaces of the cracks, which promotes hydration of unhydrated cement particles, thus sealing the crack [21,22]. Thus, SAPs show potential for use as self-healing agents in mortar and concrete.

SAPs in concrete also has some disadvantages [23,24]. One of the main drawbacks is that SAPs absorb mixing water during the preparation and casting of mortar or concrete, leading to reduced workability [25]. Second, SAPs leave large holes in the hardened concrete after losing water, resulting in decreased strength [26]. When the amount of SAPs reached 5–13% of cement weight, the compressive strength decreased by 80–87% [23]. Therefore, to alleviate these negative effects, SAPs are usually preabsorbed and the incorporated amount is <0.6% [27]. However, SAPs can ensure self-healing of concrete only when their content is >1% [8,28]. Moreover, higher SAP content accelerates the speed of self-healing of concrete [29]. Therefore, two methods have been reported to modify SAPs for use in self-healing of concrete for overcoming their deteriorating effect on the workability of concrete. In one method, pH-responsive SAPs are synthesized that will not absorb water and swell under the highly alkaline environment during concrete mixing [30,31]. In the other method, SAPs are coated using a fluid bed spraying process, which considerably delays swelling for a short time during concrete mixing [32].

Self-healing concrete shows great potential in infrastructures through reduction of maintenance and repair in the use phase. To realize the goal of engineering application, the robustness of self-healing materials should be considered in the research [33]. Concrete structures are exposed to a variety of environments, such as continuously dry, continuously wet, or alternately dry and wet; therefore, self-healing approaches should be versatile to allow recovery of material properties in widely varying environments. Self-healing approaches are also expected to function multiple times over the lifetime of the structures [34].

Herein, we propose novel SAP microcapsules for self-healing of concrete; in this method, calcium alginate is used as the shell of the microcapsule and epoxy resin is used as the liquid core. The core plays two roles: one, it reduces the water absorption and swelling rate of the microcapsules [35], thereby mitigating the disadvantages of SAPs in the workability of concrete, and two, it acts as a self-healing unit that heals cracks under dry conditions. When cracks occur, the core flows out and bonds the crack under anhydrous conditions, while the shell absorbs water under wet conditions for self-sealing and promoting the autogenous healing of cracks. Thus, the self-healing of the microcapsules is extended to adapt to dry and wet environments. In case of microcapsules, research should focus on whether the absorption of the shell causes the workability of concrete to deteriorate, whether it still has the internal curing effect to reduce the shrinkage of the concrete, and its influence on the self-healing of the concrete.

In this paper, SAP microcapsules with calcium alginate shells and epoxy resin cores were prepared. Dry microcapsules were added to cement mortar, and the fluidity of paste, autogenous shrinkage, dry shrinkage, and strength of mortar were investigated. The recovery of impermeability and compressive strength of the mortars was also tested to evaluate the self-healing effect.

## 2. Materials and Methods

### 2.1. Materials

In this study, P∙I 42.5 Portland cement was used (Qufu Zhonglian Cement Co., Ltd., Qufu, China). The density of cement clinker was 3.15 kg/m^3^, and the specific surface area was 347 m^2^/kg. The chemical and mineral compositions of Portland cement are shown in Table 1 and Table 2. The sand used in the experiments was ISO standard sand (GB/T 17671) produced by Xiamen Europe Standard Sand Co., Ltd. (Xiamen, China). The ISO particle size distribution of standard sand is shown in Table 3. PCE was used as the admixture for mortar (molecular weight between 30,000 and 50,000, produced by China building materials Zhongyan Technology Co., Ltd. (Beijing, China)); its effective solid content was 45%, and the water reduction rate was 25%. The water used in experiments, unless specifically indicated, was Beijing tap water. Raw materials used for microcapsule preparation are listed in Table 4.

### 2.2. Specimens

#### 2.2.1. Preparation of Microcapsules

**Figure 1:**
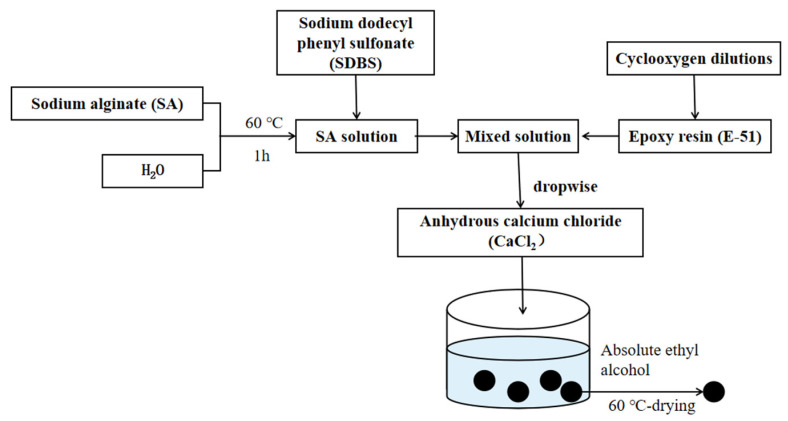
Schematic representation of microcapsule preparation process.

Calcium alginate-epoxy microcapsules were synthesized using the sharp hole-solidification bath method [35]. The synthesis process is illustrated in Figure 1.

Deionized water and sodium alginate (1.6% of deionized water weight) were added to four 500-mL flasks, placed in a 60 °C thermostatic water bath, and mixed at 400 rpm for 1 h. Then, sodium dodecyl phenyl sulfonate (1.25% of epoxy weight) was added to these mixtures. Thereafter, specific weights of E-51 epoxy resin (3.5, 5.5, and 7.5 times the weight of sodium alginate) and diluent (1.5% of epoxy weight) were added to these sodium alginate mixtures and mixed for 1 h to ensure good dispersion. Thereafter, the mixture was dripped into a calcium chloride solution through an orifice mold. The mixture was allowed to stand for 2.5 h for solidification, and then, the microcapsules were washed with anhydrous ethanol and dried at 60 °C. The resulting microcapsules had a calcium alginate outer shell, a net-like structure inside, and epoxy was filled in the cavities [35]. The core content and water absorption of microcapsules with different ratios of sodium alginate and epoxy are shown in Table 5.

#### 2.2.2. Preparation of Mortar Specimens

The mortar was prepared with a cement-to-water ratio of 1:0.29 and cement-to-sand ratio of 1:3, and the amount of PCE added was 0.2% by cement weight. The epoxy curing agent was added to the mortar at 15% of weight of microcapsules. The microcapsules added in all experiments were dry and not preabsorbed.

### 2.3. Testing Methods

#### 2.3.1. Three-Dimensional X-ray Computed Tomography (3D-XCT)

The cement paste specimens containing microcapsules were prepared with a water-to-cement ratio of 0.29 (dimensions: 20 mm × 20 mm × 40 mm) and cured at 55 ± 5% RH and 20 ± 2 °C. ZEISS Xradia 520 Versa (Carl Zeiss Microscopy, LLC, Peabody, MA, USA) was used to observe the microcapsules in the hardened paste and examine changes in the volumes of the microcapsules with different hydration durations.

#### 2.3.2. Fluidity of Paste

The spreading diameter of paste was tested according to the method of GB/T 8077-2012 as per the national standards of the People’s Republic of China. The water-to-cement ratio of paste was 0.29. A flow cone (R1 = 36 mm, R2 = 60 mm, H = 60 mm), as specified in Chinese standard GB 8077-2012, was filled with the paste. After removing the cone and waiting for 30 s, the maximum diameter of spread and the maximum width perpendicular to that diameter were measured. The average of these two values was defined as the flow value.

#### 2.3.3. Compressive and Flexural Strength of Mortar

Using the obtained mortar, three specimens were molded (dimensions of 40 mm × 40 mm × 160 mm) and cured under standard conditions (20 °C ± 2 °C, ≥95% RH) for 24 h. The mortar was demolded after 24 h and moved to a curing room (20 °C ± 5 °C, 55% ± 5% RH) and cured for 1, 3, 7, and 28 days. After curing for the specified number of days, the compressive strength and flexural strength of mortar were tested according to the method of GB/T 17671-1999 in the national standards of the People’s Republic of China. Each specimen was divided into two for compressive strength measurements. Three and six replicates were measured for flexural and compressive strength tests, respectively.

#### 2.3.4. Autogenous Shrinkage of Mortar

The instrument used in this experiment was developed as per American standard ASTM C1698-09. Each group had three parallel samples and the average was the final result. The mortar was prepared with a cement-to-water ratio of 1:0.29 and a cement-to-sand ratio of 1:2, and weight of PCE added was adjusted according to the spreading diameter of mortar of 130–140 mm. Autogenous shrinkage was tested using a vertically placed plastic corrugated tube (diameter = 29 mm, length = 430 mm), and its bottom was sealed with a special sealing plug. For every 5 cm height of pouring, the mortar was vibrated on a vibrating table for 30 s to remove trapped air. After pouring, the corrugated tube was covered with a sealing plug, and both ends of the tube were wrapped with plastic wrap to isolate the moisture and heat exchange from the outside. Then, the corrugated tube was placed in a length measuring instrument to measure the values of changes of length for 7 days.

#### 2.3.5. Dry Shrinkage of Mortar

Mortar was prepared and cured according to the standard method of JC/T 603-2004 in the industrial recommendation standards of the People’s Republic of China. Each group had three parallel samples and the average was the final result. For the specified water-to-cement ratio of 0.29 and cement-to-sand ratio of 1:2, the amount of PCE added was adjusted according to the spreading diameter of mortar (130–140 mm), and the amount of curing agent added was 15% of the microcapsules weight. The mortar was placed in a mold (dimensions: 25 mm × 25 mm × 280 mm). The specimens were cured under standard conditions (20 °C ± 2 °C, ≥95% RH) for 24 ± 2 h. Then, they were taken out and stored in water for 48 h inside a constant temperature chamber at 25 °C. They were removed from the water and subjected to drying shrinkage tests under the conditions of 20 ± 3 °C and a relative humidity of 50% ± 4%. After measuring the initial length of the specimens, they were placed in a length comparator to measure the changes in lengths for 28 days. The shrinkage of the mortar was calculated as the ratio of the change in the length of the mortar test block to the initial length.

The drying shrinkage rate of mortar was calculated according to Equation (1). The calculated value was accurate to 0.001%.

(1) $$S_{n}=\frac{\left(L_{0}-L_{n}\right)\times 100}{280}$$

where *S_n_*—Dry shrinkage rate of cement mortar after aging for *n* days, %; *L*_0_—Initial measuring length of mortar, mm; *L_n_*—Length of mortar measured at *n* days, mm; 280—Length of the mold, mm.

#### 2.3.6. Self-Healing Performance

##### Impermeability Recovery of Self-Healing Mortar

The permeability of mortar was measured according to the method of JGJ/T 70-2009 in the industrial standards of the People’s Republic of China. The mold used was a metal truncated cone with an upper diameter of 70 mm, lower diameter of 80 mm, and height of 30 mm. Six replicate specimens were used for each group. After curing at room temperature 20 ± 5 °C for 24 ± 2 h, the specimens were demolded and then moved to the curing room for aging under specified conditions of 55 ± 5% RH and temperature of 20 ± 2 °C. Finally, the specimens were sealed and installed in the mortar penetrometer for the permeability tests. The initial test pressure was 0.2 MPa, and after 2 h, it was increased to 0.3 MPa, lasting for 1 h, and then increased by 0.1 MPa every hour. The test continued until there was water seepage on the terminal faces of 3 out of 6 specimens. The impermeability coefficient of the mortar test specimens was calculated using Equation (2).

(2) $$I=\sum P_{i}\cdot T_{i}$$

where *I*—Impermeability value of mortar, MPa·h; *P_i_*—Water pressure at each pressure stage, MPa; *T_i_*—Duration of the corresponding pressure stage, h.

##### Mechanical Recovery of Self-Healing Mortar

Self-healing mortar was prepared based on the method described in Section 2.3.3. After hydration for 28 days, compressive experiments were used to create cracks. To investigate the mechanical recovery of self-healing mortar, the samples were pre-cracked by applying 40% of maximum pressure (f_max_). Subsequently, the samples were placed in a curing room (20 °C ± 5 °C, ≥95% RH) for 14 days. The compressive strength of healed samples was tested again to evaluate the self-healing performances. The self-healing rate of mechanical strength was calculated using Equation (3).

(3) $$M_{h}=\frac{R_{hc}}{R_{c}}\;\times \;100\%$$

where *M_h_*—Compressive strength recovery, %; *R_hc_*—Compressive strength after healing, MPa; *R_c_*—Initial compressive strength, MPa.

## 3. Results and Discussion

### 3.1. Volume Change of SAP Microcapsules in Hardened Paste

**Figure 2:**
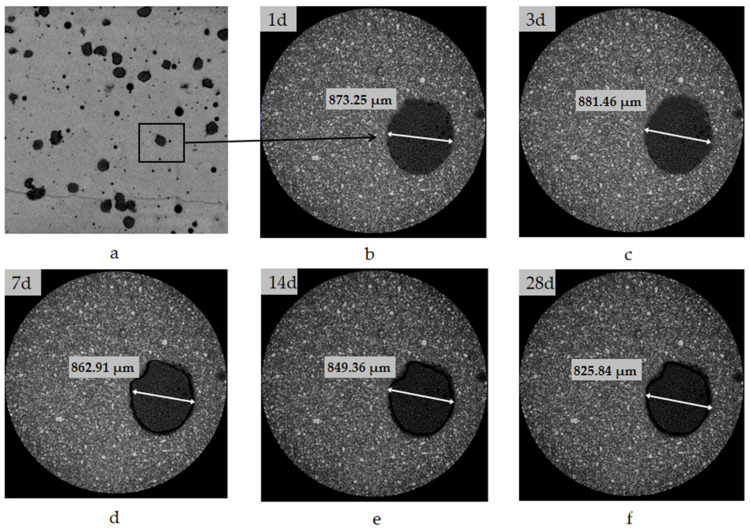
Volume change of microcapsules in hardened paste. (**a**) Hardened cement paste containing microcapsules, aged for (**b**) 1 day, (**c**) 3 days, (**d**) 7 days, (**e**) 14 days, and (**f**) 28 days.

Figure 2 shows the volume change of SAP microcapsules in hardened cement paste caused by the release of water during the hydration process.

In Figure 2, the dark-colored dots represent microcapsules dispersed in the matrix. The same microcapsule at different hydration ages is observed in Figure 2b–f. The size of microcapsule was maximum at 3 days of aging (Figure 2c). This phenomenon indicates that the microcapsules in the paste absorbed water and swelled, and this process lasted for several days with a water-cement ratio of 0.29. This slow absorption by the microcapsules mitigated the negative impact of SAPs on the fluidity of the mortar.

With continued hydration, the water in the paste continued to be consumed, decreasing the internal humidity of the paste, and the microcapsules decreased in size because of release of water. This process lasted for 28 days. The long-term release of water from the microcapsules was beneficial for reducing the drying shrinkage of mortar.

The microcapsules absorbed and lost water much longer and slower than SAPs, which would be beneficial for the workability, strength, and dry shrinkage reduction of mortar or concrete.

### 3.2. Effect of SAP Microcapsules on the Fluidity of Cement Paste

**Figure 3:**
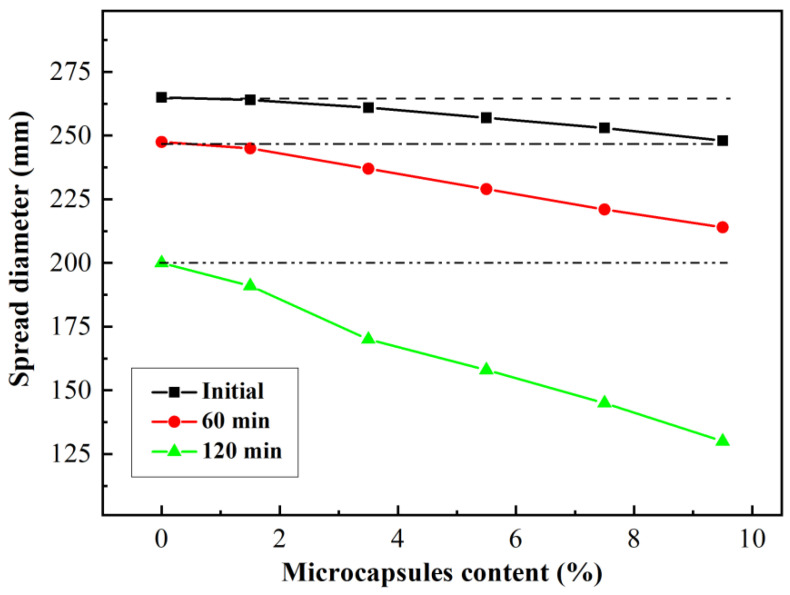
Fluidity of cement paste with different amounts of microcapsules added.

Dry microcapsules with a core content of 73.4% and an average particle size of 0.7 mm were added to the cement pastes at 1.5%, 3.5%, 5.5%, 7.5%, and 9.5% of cement weight. The effect of microcapsules on the fluidity of pastes is presented in Figure 3.

Figure 3 shows the decreasing fluidity of the pastes from the starting of the experiment, at 60 min and at 120 min, with increasing microcapsule content; the influence of microcapsules on fluidity increased with time. This phenomenon is attributed to the water absorption of the microcapsules. As the time prolonged, the amount of water absorbed by the microcapsules increased, resulting in a decrease in the fluidity of paste. This phenomenon also occurred when the content of microcapsules increased.

When the microcapsule content was 3.5%, compared with the reference group, the loss of the fluidity of pastes was 1.5%, 4.2%, and 15% at the start of the experiment, at 60 min, and at 120 min, respectively. The effect of the microcapsules on the fluidity of paste was delayed, which indicated that the water absorption rate of SAPs decreased on incorporation into microcapsules. This effect is attributed to the inhibition of water absorption by the hydrophobic polymer in the core. Therefore, it can be inferred that the influence of the microcapsules on the workability of the mortar or concrete was considerably weakened.

### 3.3. Effect of SAP Microcapsules on the Compressive and Flexural Strengths of Mortar

**Figure 4:**
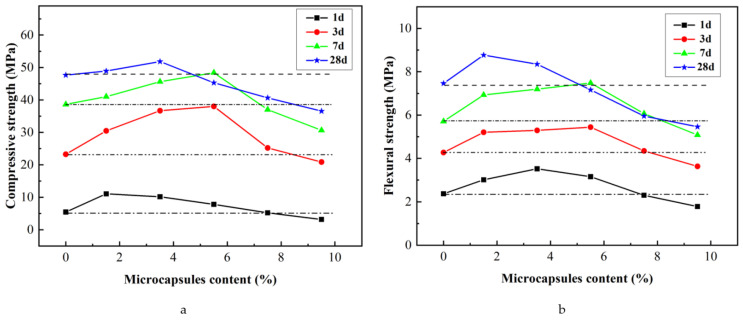
Effect of microcapsule content on compressive and flexural strengths of mortars. (**a**) Compressive strength, (**b**) flexural strength.

The microcapsules added to the mortars were the same as those in Section 3.2. The compressive and flexural strengths of mortars are shown in Figure 4.

Figure 4 shows the experimental results when microcapsules content was <7.5%; the compressive and flexural strengths of mortars at 3, 7, and 28 days were higher than those of the reference groups. The microcapsules released water during this period, because of which the interior of the mortar maintained a high relative humidity, promoting cement hydration. In particular, because of the low water-to-cement ratio of 0.29 and the dry curing conditions (55 ± 5% RH), the internal curing effect was strengthened. This is conducive to the development of the strength of mortar, resulting in the strength of the mortar containing microcapsules at 3 d and 7 d higher than that of the reference group. In addition, the experimental results indicated that the loss of water from the microcapsules did not have a significant effect on the strength of the hardened mortar.

When microcapsules content was >7.5%, the compressive and flexural strengths of mortar were lower than those of the reference groups. This was because the strength of the microcapsules was lower than that of mortar. As microcapsules content increased, the strength of mortars decreased.

### 3.4. Effect of SAP Microcapsules on the Autogenous Shrinkage and Drying Shrinkage of Mortar

#### 3.4.1. Effect of Microcapsules on Autogenous Shrinkage of Mortar

**Figure 5:**
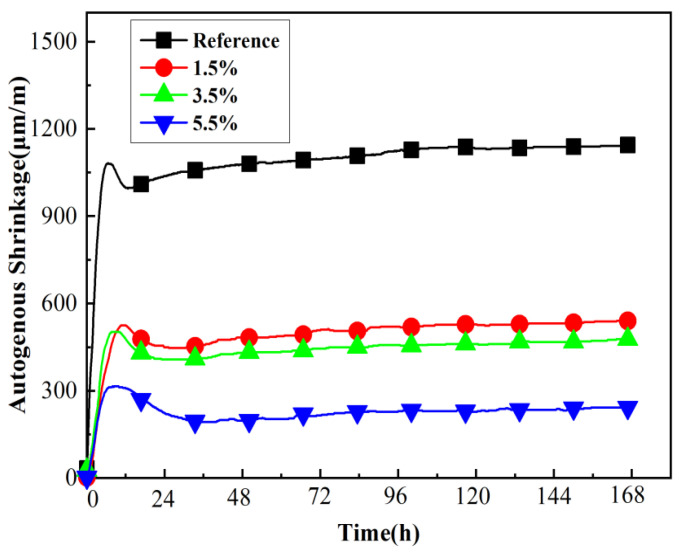
Autogenous shrinkage of mortars with different microcapsule content.

Dry microcapsules with a core content of 73.4% and an average particle size of 0.7 mm were added to the mortars at 1.5%, 3.5%, and 5.5% of cement weight. The autogenous shrinkage of mortars is presented in Figure 5.

Figure 5 shows two stages of autogenous shrinkage of the cement, which is consistent with the typical data of silicate cement continuity autogenous shrinkage measured by Lura et al. [36]. Compared with the reference group, the addition of SAP microcapsules could effectively mitigate the autogenous shrinkage of the mortar in early age, and this effect increased with increase in microcapsules content. The experimental results indicated that the microcapsules had an internal curing effect. The microcapsules released water to maintain the internal humidity of the mortar, that is, reduce the self-drying effect caused by cement hydration, thereby reducing the autogenous shrinkage.

**Figure 6:**
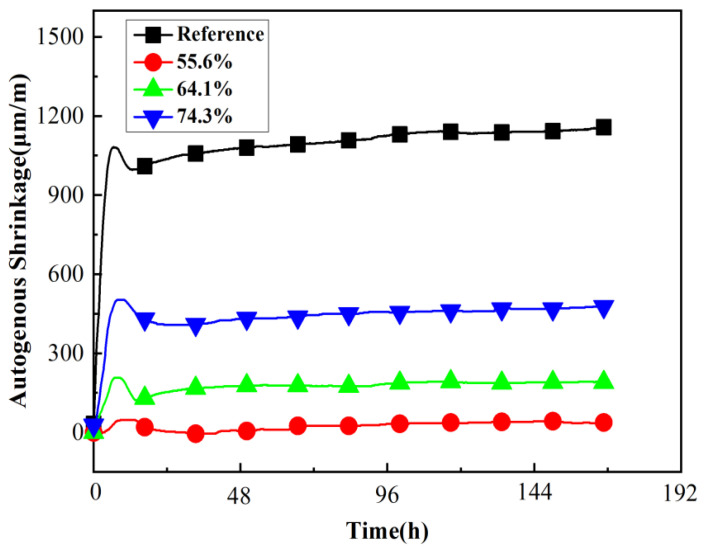
Autogenous shrinkage of mortars with different core content microcapsules.

The experimental results of the autogenous shrinkage of mortars containing microcapsules with different core contents are shown in Figure 6. The microcapsules content was 3.5% of cement weight, and the core content of the microcapsules was 55.6%, 64.1%, and 74.3%, respectively.

Figure 6 shows that as the core content of the microcapsules decreased, the autogenous shrinkage of mortar decreased. This was because of the internal curing effect of microcapsules, which was enhanced by the higher SAP content. Furthermore, on addition of microcapsules with a core content of 55.6% and 3.5% of cement weight, the autogenous shrinkage of the mortar was almost eliminated. This indicates that addition of higher microcapsules content can compensate for the weakened water absorption of SAPs.

#### 3.4.2. Effect of SAP Microcapsules on the Drying Shrinkage of Mortar

**Figure 7:**
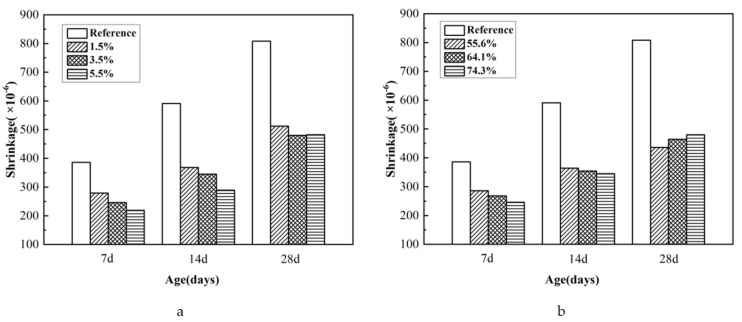
Influence of microcapsule the drying shrinkage of mortar. (**a**) Different microcapsules content, (**b**) different core content.

The experimental results of the drying shrinkage of mortars containing microcapsules are shown in Figure 7. In Figure 7a, the core content of the microcapsules was 74.3%, and microcapsules content was 0%, 1.5%, 3.5%, and 5.5%. In Figure 7b, microcapsules content was 3.5% of cement weight, and the core content of the microcapsules was 55.6%, 64.1%, and 74.3%.

As shown in Figure 7, the addition of microcapsules effectively reduced the drying shrinkage of mortar. As shown in Figure 7a, the reduction of drying shrinkage increased with increasing microcapsules content. This was attributed to the strengthening of internal curing because the microcapsules distributed in a unit volume of mortar increased with increasing microcapsules content, which led to release of more water, exerting a stronger inhibitory effect on drying shrinkage [37].

Figure 7b shows that compared with autogenous shrinkage, the inhibition of drying shrinkage was not sensitive to the core content of microcapsules. It was presumed that the change in internal curing caused by the difference in the core content of microcapsules was not sufficient to significantly influence the drying shrinkage.

### 3.5. Self-Healing Effect of SAP Microcapsules

#### 3.5.1. Impermeability Recovery of Self-Healing Mortar

**Figure 8:**
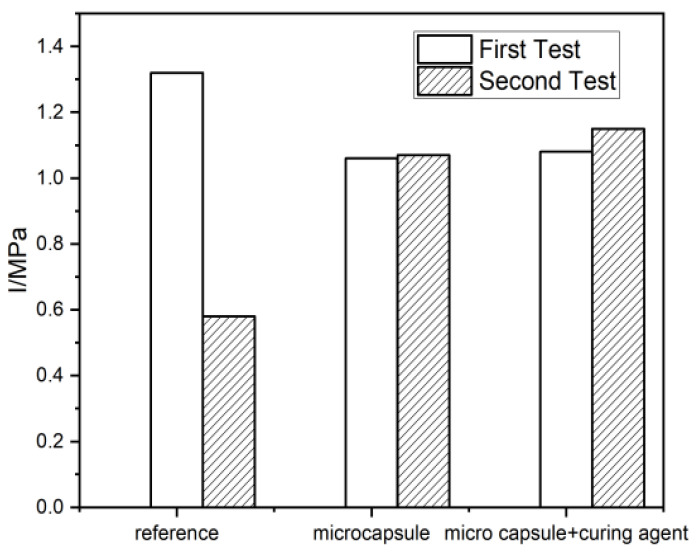
Impermeability of the mortars in two tests.

Microcapsules with a core content of 74.3% were added to the mortar at 3.5% of cement weight, and tetraethylenepentamine was added as epoxy hardener, 0% and 15% by weight of the microcapsules. After the specimens were cured for 28 d under dry conditions, the first impermeability test was performed; the second impermeability test was carried out after seven dry and wet cycles for 14 d. The results of the two impermeability tests are shown in Figure 8.

As shown in Figure 8, the impermeability of the mortars containing microcapsules decreased compared with the reference group in the first test and that the addition of epoxy hardener in the mortar had no effect on the impermeability. This may be attributed to the fact that the microcapsules aggravated the internal defects of the mortar as they did not absorb enough water in a short time to swell for blocking the water channels.

In the second test, the impermeability of mortar containing microcapsules was well-maintained and the impermeability of the mortar with curing agent was increased, while the impermeability of the reference group was significantly reduced. This is attributed to the self-healing effect of the microcapsules. The water-absorbing microcapsules swell and block the water-permeable channel. This experimental phenomenon was demonstrated in a previous study [36]. Moreover, the SAP shells of microcapsules promote the autogenous healing of the microcracks after seven dry and wet cycles. Owing to the combined effect of the two, the impermeability of the microcapsule-containing mortars was significantly higher than that of the reference group. Furthermore, the pressure in the impermeability test caused part of the microcapsules to rupture; the outflowing epoxy reacted with the hardener under dry conditions during the dry-wet cycle, solidified, and blocked the cracks. As a result, the impermeability of the mortar was improved. Thus, the experimental results showed that the adding microcapsules can promote effective self-recovery of the impermeability of mortar.

#### 3.5.2. Mechanical Recovery of Self-Healing Mortar

**Figure 9:**
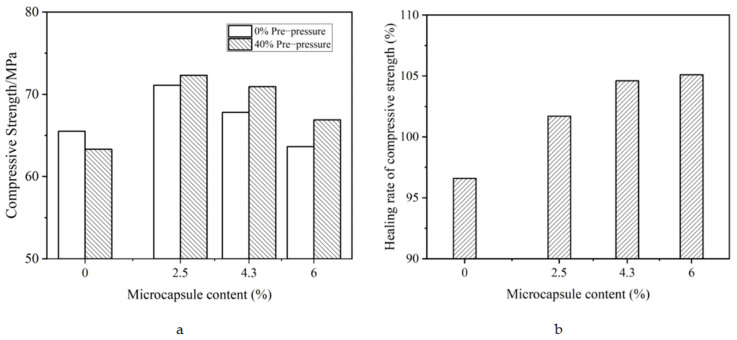
Influence of microcapsule content on the recovery of compressive strength. (**a**) Compressive strength, (**b**) the recovery of compressive strength.

Figure 9 shows the mechanical recovery of self-healing mortar after pre-cracking by applying 40% f_max_ (f_max_ = 70 kN). Pre-cracking is a popular method for the mechanical recovery investigation of self-healing cementitious materials [38].

As shown in Figure 9, when the samples were pre-cracked, the compressive strength of reference mortars decreased, while that of samples with microcapsules increased. This was attributed to the effect of the self-healing agent of the microcapsule core. The pre-cracking of the samples caused the microcapsules to rupture, and the core healing agent flowed out to bond the cracks. Therefore, the recovery of compressive strength increases with increasing microcapsule content. In addition, the pre-cracking treatment of the samples also made them denser, which was beneficial to the compressive strength. Thus, Figure 9a shows that the compressive strength of the pre-cracked healing samples was higher than that of the non-pre-cracked samples.

The recovery of mechanical property of self-healing mortar was not as obvious, especially compared with the recovery of impermeability. This was because only the core of the microcapsule played a role in the recovery of compressive strength under the experimental conditions, and both the shell and core of the microcapsule contributed to the recovery of impermeability. 

## 4. Conclusions

Absorbent microcapsules with calcium alginate as the shell and epoxy resin as the core were prepared via a sharp hole-solidification bath method. The absorption rate of microcapsules was lower and the absorption speed was slower than that of SAPs; therefore, the influence of microcapsules on the fluidity of cement paste was delayed and weakened, showing the feasibility of high content of microcapsules in the mortar. Moreover, the addition of absorbing microcapsules significantly reduced the autogenous shrinkage and drying shrinkage of the mortar. Therefore, the absorbing microcapsules not only retain the internal curing function, but also overcome the adverse effects of SAPs on the workability and strength. Most importantly, the shell and the core of the absorbing microcapsules functioned under dry and wet environments to repair the impermeability and mechanical properties, showing a synergistic effect and achieving satisfactory recovery of impermeability. However, the absorbing-releasing behavior of microcapsules may weaken the interface effect, resulting in a decrease in the probability of cracks breaking the microcapsules, which should be investigated and addressed in future studies.

## Figures and Tables

**Table 1:** Chemical compositions of Portland cement.

Composition	SiO_2_	Al_2_O_3_	Fe_2_O_3_	CaO	MgO	SO_3_	Na_2_O	f-CaO
Content/%	21.73	4.60	3.45	64.65	3.56	0.46	0.59	0.96

**Table 2:** Mineral compositions of Portland cement.

Composition	C_3_S	C_2_S	C_3_A	C_4_AF
Content/%	56.62	19.58	6.36	10.49

**Table 3:** ISO particle size distribution of standard sand.

Length of Square Hole/mm	2.0	1.6	1.0	0.5	0.16	0.08
Cumulative screening/%	0	7 ± 5	33 ± 5	67 ± 5	87 ± 5	99 ± 1

**Table 4:** Materials for preparation of microcapsules.

Drug Name	Pure Degree	Manufacturer
Sodium alginate (SA)	Solid content is 99.9%	Tianjin Institute of Guangfu Fine Chemical Industry (Tianjin, China)
Epoxy resin (E-51)	Solid content is 99.8%	Shanghai Aotun Huagong Technology Co., Ltd. (Shanghai, China)
Cyclooxygen dilutions	-	Shanghai Aotun Huagong Technology Co., Ltd. (Shanghai, China)
Sodium dodecyl phenyl sulfonate (SDBS)	AR	Tianjin Forchen Chemical Reagent Factory (Tianjin, China)
Anhydrous calcium chloride (CaCl_2_)	Solid content is 96.0%	Tianjin Forchen Chemical Reagent Factory (Tianjin, China)
Absolute ethyl alcohol	AR	Tianjin Damao Chemical Reagent Factory (Tianjin, China)

**Table 5:** Core content and water absorption of microcapsules under different raw material ratios.

Number	mE-51:mSA	Core Content/%	Water Absorption Rate/%
A	3.5	55.6	37.8
B	5.5	64.1	28.2
C	7.5	74.3	24.0

## Data Availability

All the data is available within the manuscript.
